# A Descriptive Analysis on the Impact of COVID-19 Lockdowns on Road Traffic Incidents in Sydney, Australia

**DOI:** 10.3390/ijerph182111701

**Published:** 2021-11-07

**Authors:** Sai Chand, Ernest Yee, Abdulmajeed Alsultan, Vinayak V. Dixit

**Affiliations:** 1 Research Centre for Integrated Transport Innovation (rCITI), School of Civil and Environmental Engineering, University of New South Wales, Sydney 2052, NSW, Australia; v.dixit@unsw.edu.au; 2 School of Civil and Environmental Engineering, University of New South Wales, Sydney 2052, NSW, Australia; ernestpyee08@gmail.com; 3 Department of Civil Engineering, College of Engineering, Prince Sattam Bin Abdulaziz University, Al-Kharj 16273, Saudi Arabia

**Keywords:** COVID-19, crashes, safety, duration

## Abstract

COVID-19 has had tremendous effects worldwide, resulting in large-scale death and upheaval. An abundance of studies have shown that traffic patterns have changed worldwide as working from home has become dominant, with many facilities, restaurants and retail services being closed due to the lockdown orders. With regards to road safety, there have been several studies on the reduction in fatalities and crash frequencies and increase in crash severity during the lockdown period. However, no scientific evidence has been reported on the impact of COVID-19 lockdowns on traffic incident duration, a key metric for crash management. It is also unclear from the existing literature whether the impacts on traffic incidents are consistent across multiple lockdowns. This paper analyses the impact of two different COVID-19 lockdowns in Sydney, Australia, on traffic incident duration and frequency. During the first (31 March–28 April 2020) and second (26 June–31 August 2021) lockdowns, the number of incidents fell by 50% and 60%, respectively, in comparison to the same periods in 2018 and 2019. The proportion of incidents involving towing increased significantly during both lockdowns. The mean duration of crashes increased by 16% during the first lockdown, but the change was less significant during the subsequent lockdown. Crashes involving diversions, emergency services and towing saw an increase in the mean duration by 67%, 16%, and 47%, respectively, during the first lockdown. However, this was not reflected in the 2021 data, with only major crashes seeing a significant increase, i.e., by 58%. There was also a noticeable shift in the location of incidents, with more incidents recorded in suburban areas, away from the central business area. Our findings suggest drastic changes in incident characteristics, and these changes should be considered by policymakers in promoting a safer and more sustainable transportation network in the future.

## 1. Introduction

SARS-CoV-2, also known as the 2019 novel coronavirus (COVID-19), has had tremendous effects worldwide, resulting in large-scale death and upheaval. At the beginning of March 2020, the World Health Organization (WHO) declared it a pandemic, the first one in over 100 years since the 1918 influenza pandemic. As of September 2021, COVID-19 has claimed over 4 million lives, infected over 200 million people and is still an ongoing worldwide crisis with over 600,000 daily cases worldwide. COVID-19 has meant that governments have taken measures and introduced unprecedented policies for our time, which have had a widespread impact on the way we live our day-to-day lives. These policies have been aimed at reducing travel and mobility in large cities to combat community transmission of COVID-19. Governments have imposed varying degrees of limitations on mobility, internationally and locally.

These policies and orders by governments, also known as “lockdown” orders, restrict people from leaving their homes except for essential reasons, and office workers and students have moved to remote work or learning. An abundance of studies have shown that traffic patterns have changed worldwide as working from home has become dominant, with many facilities, restaurants and retail services being closed due to these orders. Urban traffic around the world, without any exception, has observed a drastic reduction in traffic congestion. For example, cities such as Milan, Paris, Madrid and Manchester saw a reduction in congestion by 85%, 84%, 83%, and 76%, respectively, during the lockdown period compared with the baseline scenario [1]. The reduced traffic movement has resulted in a reduction in fuel consumption [2], road crashes and fatalities [3,4,5,6] and air pollution [7,8], and an increase in travel time reliability [9]. On the other hand, lockdowns have also been associated with more frequent harsh acceleration and braking events, mobile phone usage [3], and increased crash severity [4].

Road traffic safety can be characterised by different metrics such as crash frequency, fatalities, rates, severity, and duration [10,11]. There has been much emphasis on the reduction in fatalities and crash frequencies during the lockdown period because of the relative ease in obtaining such data. There are a few studies on crash severity and rate [12], but to the best of the authors’ knowledge, there is no study on the impact of lockdown on traffic incident duration. On the one hand, decreased congestion and crash frequency could help the emergency services to reach crash locations faster, thus reducing crash duration. On the other hand, increased crash severity could increase the difficulty in clearing the incident. Therefore, assessing the net impact of lockdowns on crash duration is of interest.

Furthermore, the existing studies on the impact of COVID-19 have only focused on crashes. Other traffic incidents, such as breakdowns, may not result in as much emotional, social, and economic losses as crashes but still significantly reduce roadway capacities and operating conditions. Although vehicles’ quality and safety performance have been improved over the years, these incidents cannot be avoided entirely and continue to be a critical problem for road users, authorities, and roadside assistance companies [13,14].

Finally, it is unclear from the existing literature whether the impacts on traffic incidents were consistent across multiple lockdowns. In this context, the current paper aims to quantify the impacts of multiple COVID-19 lockdowns on road safety, specifically on incident duration and frequency in Sydney, Australia. The rest of the paper is organised as follows. First, a review of a few studies on the impacts of COVID-19 on crash characteristics is provided. This is followed by a description of the data and methodology used in this paper. Finally, the results are described and followed by the conclusions and potential future directions.

## 2. Literature Review

Current studies show consistency in regard to the fact that traffic-related incidents have decreased significantly due to stay-at-home orders, which limit mobility [3,15]. Saladié et al., (2020) highlight the impact of strict lockdowns in Tarragona province in Spain, with the reduction in crashes (74.3%) being higher than the decrease in mobility (62.9%) during that period [15]. Another study from Spain reported a 67% reduction in the number of crashes in the city of Santander [16]. A study from Greece observed a 62% reduction in crashes, a 68% reduction in fatalities and a 48% drop in serious injuries [17]. However, another study from Greece reported a 57% reduction in traffic but only a 42% reduction in crashes [18].

Muley et al. (2021) reported a 30% drop in traffic movement and a 37% reduction in crash frequency in Qatar [19]. A study conducted in Greece and Saudi Arabia showed a 6% to 11% increment in speeding violations and up to 12% increase in harsh braking events during March and April 2020 [3]. In California, researchers estimated that the crash frequency dropped by 60% [20]. In another study, researchers observed a 65% reduction in collisions during the lockdown in Toronto and a 59% reduction during the “re-opening” period [21].

A study conducted across the USA reported that minor crashes decreased by up to 23%, but the most severe crashes went up by 18% [22]. In Missouri, researchers found a significant reduction in crashes leading to minor or no injuries, but not in crashes resulting in severe or fatal injuries [6]. In the state of Ohio, USA, Li et al. (2021) observed a reduction of up to 55% in crashes, 47% in injuries, 34% in severe or fatal injuries, and 44% in traffic volume [23].

Some studies observed varying impacts of lockdowns on road safety among different sub-groups of the population. For example, Lin et al. (2021) noticed spatial heterogeneity in the reduction in crashes in the cities of Los Angeles and New York. They observed that the proportion of crashes increased for “Hispanic” and “Male” groups, and the crash hot spots shifted from higher-income areas to lower-income areas [24]. A study in West Virginia evaluated the crashes involving injury across different age groups and gender and observed 44% fewer injuries overall [25]. Rapoport et al. (2021) noticed a reduction in injuries by 64.7% among older drivers (aged 80 years and above) and 22.9% among middle-aged drivers (35−54 years) in Ontario, Canada. However, they did not find any significant changes among drivers of other age groups [26].

A few researchers also studied the impact of lockdowns on crash rate (i.e., crash frequency divided by traffic volume), a standardised measure of relative safety on roadway segments that is more easily interpretable than crash frequency [11,27]. For example, Doucette et al., (2020) found that single-vehicle crash rates increased 2.29 times, and single-vehicle fatal crash rates increased 4.10 times during the initial weeks of the stay-at-home periods in Connecticut, USA [12]. However, in a separate study using an extended dataset, researchers found that crash rates returned to average during the post-stay-at-home order [28].

As evident from the above review, there has been much emphasis on evaluating the impacts of COVID-19 lockdowns on crash frequency and fatalities. There are also a few studies on crash rate and severity. It is interesting to note that most studies report a crash frequency reduction in the range of 50–60%, apart from a few exceptions. Furthermore, most research has been done in European and North American countries.

Research studies on various traffic incident characteristics are still being conducted in different areas of the world at varying degrees of lockdown. At the time of writing, there is no single study on the impact of COVID-19 on crash duration and other types of incidents such as vehicle breakdowns. Since many countries have implemented multiple lockdowns, the consistency of their impact on road safety has not yet been fully evaluated.

## 3. Data and Methods

### 3.1. Area of Study

**Figure 1:**
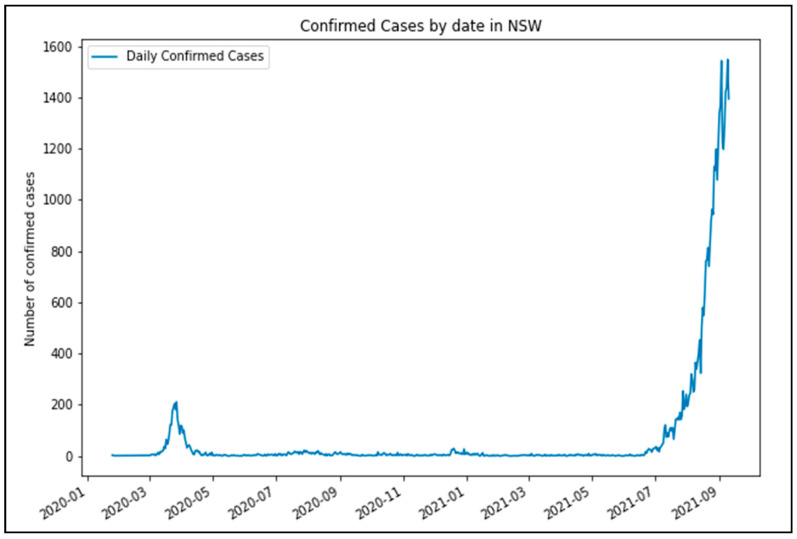
Confirmed COVID-19 cases from January 2020 to August 2021.

The study area for the analysis is Sydney, the capital of the state of New South Wales (NSW) in Australia. Sydney has a population of more than 5 million and is the most populous city in entire Oceania. Following outbreaks of COVID-19, the first lockdown period for NSW began on 31 March 2020. The extent of this lockdown order restricted residents of Greater Sydney from travelling except for essential purposes such as grocery shopping, work and education (if a person is unable to work or learn at their residence), compassionate grounds, or any other essential services. This order was loosened beginning on the 28 April 2020, where up to 2 visitors were allowed. On the 10 May 2020, cafés and restaurants were allowed to seat up to 10 people, and outdoor gatherings of up to 10 people were also allowed. Figure 1 highlights the onset of COVID-19 cases in NSW, which necessitated restrictions to stop the community transmission of COVID-19 [29].

More recently, from the last week of June 2021, a more restrictive lockdown began to be implemented with the spread of the Delta variant of COVID-19, which has been 97% more transmissible than the original COVID-19 strain [30]. It brought record infection rates in NSW, and consequently, stricter mobility restrictions were imposed. These included the same restrictions as in the first lockdown in 2020 and various other measures over two months from the 26 June 2021 to the current time of writing (early September). Some examples of restrictions that were implemented at various stages of this lockdown include:

- ***Mobility:*** Individuals were limited to a travel zone of 10 km from their residence. It was subsequentially decreased to 5 km.
- People from “high-risk” Local Government Areas (LGAs) were restricted from leaving their LGA unless they were authorised workers. This was constantly revised and added upon as new areas became COVID-19 hotspots.
- ***Public transport:*** Changed to Sunday timetables.
- ***Work:*** Construction work was paused for 2 weeks (19 July 2021 to 30 July 2021).

Initially, the restrictions were limited to the city of Sydney. However, on the 14 August 2021, statewide restrictions were made to contain the spread of COVID-19.

### 3.2. Crash Fatality Data

The data on crash fatalities were obtained from the Australian Road Deaths Database (ARDD) [31], which contains all known fatalities recorded in Australia. As of the time of writing, the data on fatalities were correct to August 2021.

The following information was available for each fatality record:

- **Time and Date**
- **Location:** State, Local Government Area (LGA)
- **Heavy Vehicle/Bus/Truck Involvement**
- **Road information:** Speed limit and type of road
- **Fatality information:** Road user, gender and age

### 3.3. Traffic Incident Data

The data on traffic incidents were obtained from the Transport for New South Wales (TfNSW) live traffic website from 1 January 2018 to 31 August 2021, a total of more than 3.5 years of records [32]. The website is managed by the NSW Transport Management Centre (TMC). It is integral in providing monitoring, communication, and traffic management systems to respond to and clear traffic incidents and provide accurate and up-to-date information for travellers on the NSW road network.

The following information is available for each traffic record:

- **Date:** Day, month, and year
- **Incident Start Time:** Recorded time of the incident
- **Incident End Time:** The time each record was cleared
- **Category:** Breakdown, crash and others as provided by the TfNSW
- **Subcategory:** Types of vehicles and number of vehicles
- **Attending groups:** Emergency services, TfNSW, tow trucks, mechanics, motorway crews, etc.
- **Is the incident major:** 1 for incident tagged major, 0 otherwise (a proxy for crash severity)
- **Diversions:** TMC recommendations for any diversions
- **Advice:** TMC advice recommendations for travellers
- **Spatial:** Latitude, longitude, suburb and road

**Figure 2:**
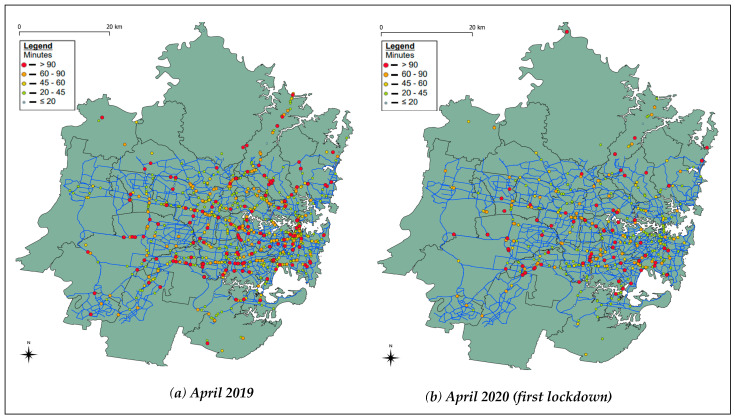
The comparison of incidents (breakdowns and crashes) in Sydney, colour-coded by duration. (**a**) During April 2019 and (**b**) during April 2020 (first lockdown).

**Figure 3:**
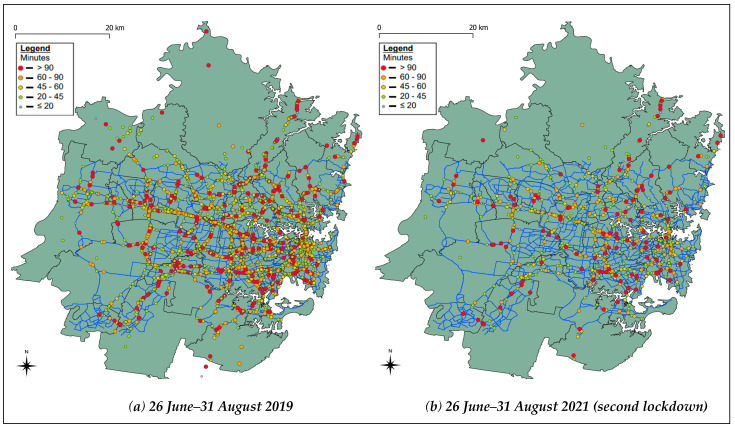
The comparison of incidents (breakdowns and crashes) in Sydney, colour-coded by duration. (**a**) During the period 26 June–31 August 2019 and (**b**) 26 June–31 August 2021 (second lockdown).

The frequency, severity and duration of incidents in the four weeks during the April 2020 strict lockdown was compared to the same period in 2019 and 2018. Similarly, the incident characteristics during the second (and stricter) lockdown during 26 June–31 August 2021 were compared with the corresponding period from 2018 and 2019. Figure 2 and Figure 3 show the comparison of incidents (breakdowns and crashes) in Sydney during both the lockdowns with the same period from previous years (pre-COVID), colour-coded by duration.

## 4. Methodology

Traffic congestion was reduced by close to 40% in metropolitan Sydney during the first lockdown and by up to 75% during the second lockdown, according to the TomTom traffic index [33]. We evaluated whether there was a similar effect on the number of fatalities and other crash characteristics, such as crash frequency, duration and severity. As we had no access to severity indicators such as injuries, proxies for severity were extracted from the dataset. These proxies include the involvement of heavy vehicles, towing, emergency services, and traffic diversions, which are often correlated with more severe crashes [34,35,36,37]. Incident duration is also correlated with severity, as more severe incidents take longer to clear [36,38]. A descriptive analysis of the impact of COVID-19 on fatalities, frequency and incident duration is provided, comparing April 2020 with the same period during April 2018 and 2019 and by comparing the data from the second lockdown with the corresponding period in 2018 and 2019.

Like Doucette (2021) and Saladié (2020), we analysed the incident ratios for each incident category and tested for statistical significance across these time periods using a Chi-square test. Furthermore, we assessed the impact of COVID-19 lockdown orders on incident characteristics and the proportion of incidents involving heavy vehicles (trucks, buses, and trailers), incidents involving towing, fatal crashes and incidents labelled as major by Transport for NSW’s Traffic Management Centre. The mean incident duration for each period was also analysed for different categories of incidents such as crashes and breakdowns, with an independent two-way t-test. All statistical tests were conducted with a statistical significance value of 0.05.

## 5. Results

### 5.1. Fatalities

A comparison of the fatalities reported for April 2020 compared to the same period in 2019 and 2018 (Table 1) reveals a 30% decrease on average. While the sample size is small, this 30% decrease in fatalities compared to a 50% decrease in crash frequency during the same period (refer to Section 5.2) highlights that the impact on fatalities was not the same as the number of crashes. Similarly, fatalities were reduced by 53% during the second lockdown, compared to the 64% reduction in overall crashes.

As can be seen in Table 1, the sample size is small and therefore, statistical tests could not be conducted to test whether the changes are significant or not. However, we can see that there has been an increase in the proportion of fatalities that have occurred on highways, and this is represented through the shift in traffic and incidents from Sydney’s Central Business District (CBD) to highway roads, as seen in Figure 2 and Figure 3. We also see that the proportion of fatalities involving a heavy vehicle (bus, truck or articulated vehicle) has increased. Furthermore, the proportion of fatalities involving a single vehicle has increased, a finding consistent with a past study [12]. However, the proportion of fatalities involving pedestrians and multiple vehicles has been reduced.

### 5.2. Incident Frequency

**Figure 4:**
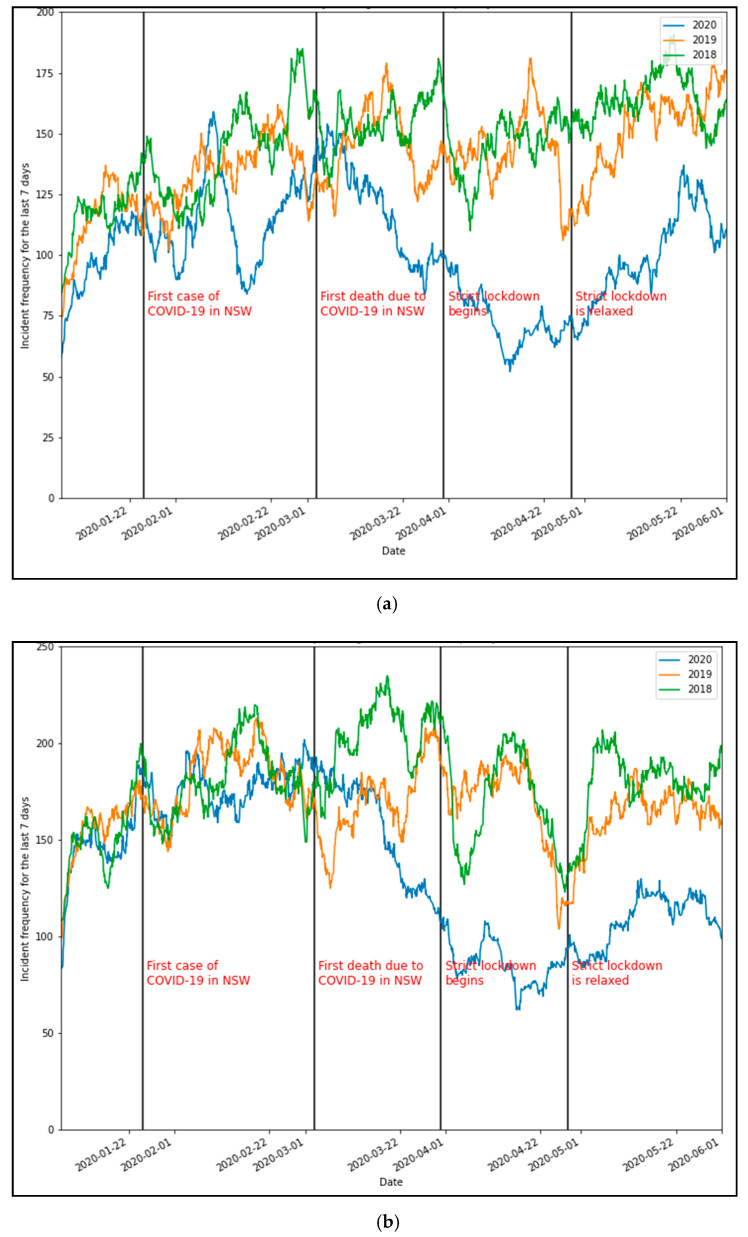
Comparison of seven-day rolling traffic incident frequency (2020 vs. 2018 and 2019). (**a**) Crash frequency and (**b**) breakdown frequency.

A comparison of incident frequency highlights a significant decrease in crashes and vehicle breakdowns during lockdown periods. Figure 4a,b showcase the overall trends in the frequency of crashes and breakdowns with important dates (during and close to the first lockdown) relating to COVID-19 highlighted. There were significant decreases following the spread of COVID-19 throughout the community as national attention began to focus on the first recorded death on the 3 March 2020. Following this, the NSW government began to enact policies and orders aimed at stopping the spread. A strict lockdown commenced on the 31 March, and the significant decline in traffic crashes continued to a nearly 60% decrease compared to peak traffic incidents in 2019. Similarly, vehicle breakdowns dropped by 50%. A comparison of the same period in 2019 and 2018 is provided to highlight these drops.

**Figure 5:**
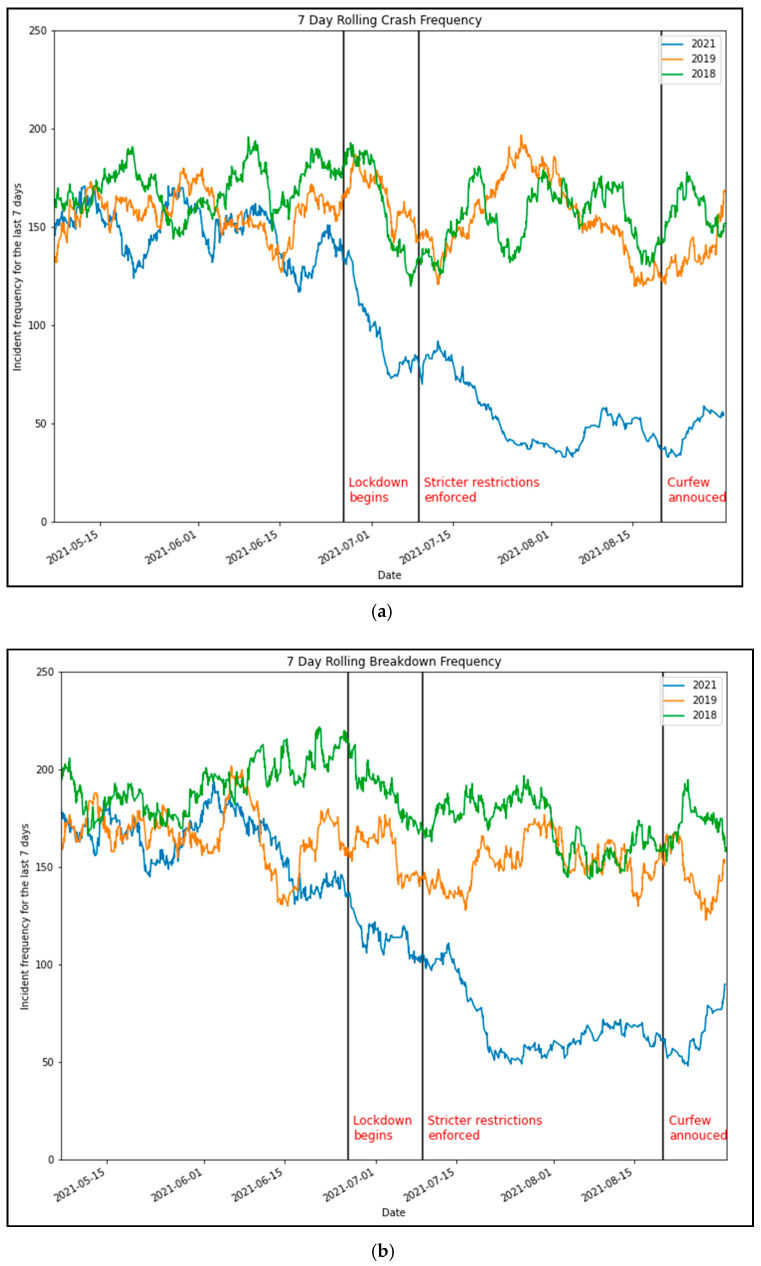
Comparison of seven-day rolling traffic incident frequency (2021 vs 2018 and 2019). (**a**) Crash frequency and (**b**) breakdown frequency.

Figure 5a,b show a similar decrease in incident frequency when the second major lockdown was announced in Sydney in late June 2021. However, we see that traffic crashes decreased by more than 70% at the peak due to the stricter restrictions in place. Similarly, vehicle breakdowns dropped by 58%.

Table 2 outlines the changes in incident frequency and characteristics and the changes in incident makeup. For each incident involving a certain characteristic, the absolute numbers and proportion relative to the total incidents recorded are presented. The incident rate ratio (IRR) of the incident types during the first and second lockdowns compared to the corresponding periods in 2019 and 2018 also highlight changes for certain indicators of severity. A total of 598 reported incidents that were labelled as breakdowns or crashes occurred during the first lockdown. The proportion of breakdowns and crashes did not change significantly, with IRR values of 1.02 and 0.98, respectively.

The proportion of crashes resulting in fatalities increased during this period, with an IRR of 1.4; however, this was not statistically significant (χ^2^ = 0.60). The proportion of crashes causing diversions increased significantly, with an IRR of 1.78, which was statistically significant (χ^2^ = 3.85, *p* < 0.01). Diversions required for incidents were associated with an increase in incident duration by up to 60% [36], which could represent a subsequent increase in severity or more directly cause traffic delays. Crashes involving bicycles saw an increase in proportion of incidents by 1.48; however, this was not statistically significant.

The presence of heavy vehicles such as buses, trucks, and articulated vehicles saw a proportionally similar uptick, with the IRR of heavy vehicle crashes and breakdowns increasing to 1.27 (χ^2^ = 2.91, *p* < 0.1) and 1.06 (χ^2^ = 0.46), respectively. This would be expected as trucks are a critical part of the NSW economy, transporting goods and various materials to and from Sydney. Despite a lockdown, any effects on heavy vehicle mobility would not be as significant as the impacts on light vehicles, given that lockdown restrictions did not impact their movement. Instead, Freight Australia reported that freight vehicles saw increased activity in a critical effort to keep supply chains open and ensure that communities were adequately supplied [39]. With most heavy vehicles travelling on high-speed highways, this could further increase the severity and fatalities from incidents [40]. The proportion of crashes that required towing also occurred at an increased rate, with an IRR of 1.60 (χ^2^ = 4.57, *p* < 0.05), with the result being statistically significant. Once again, crashes requiring towing have been shown to increase the average incident duration, and subsequently, they could be a proxy indicator for an increase in severity [36].

Table 3 outlines the same changes for the second lockdown in Sydney. A total of 1208 reported incidents that were labelled as breakdowns or crashes occurred in this lockdown period. With different measures undertaken during this period, we also saw different incident characteristics. Most notably, the composition of incidents labelled as crashes saw a statistically significant decrease from a proportion of 0.51 in 2019/2018 to 0.42 (χ^2^ = 29.94, *p* < 0.01). Furthermore, both crashes and breakdowns involving towing saw a statistically significant increase in their incident rate (χ^2^ = 33.46, χ^2^ = 9.61, *p* < 0.01). Similarly, crashes involving bicycles saw a significant uptick in incident rate; however, unlike 2020 this was statistically significant.

However, unlike the 2020 lockdown, there was no statistically significant change in the incident rates for crashes or breakdowns involving heavy vehicles. We could attribute this to the different rules and restrictions put in place as COVID-19 cases began escalate rapidly. Some of the most notable restrictions that would have contributed to this decrease were the 2-week pause on construction work from 19 July to 30 July 2021, and the capacity reduction on construction sites to 50% maximum capacity [41]. A change in public transit to weekend timetables from the 23 August would consequently reduce bus movement [42]. There have also been reports of agitation, protests and delays in the road freight industry due to regular changes in border rules and COVID-19 testing requirements [43,44].

### 5.3. Incident Duration

**Figure 6:**
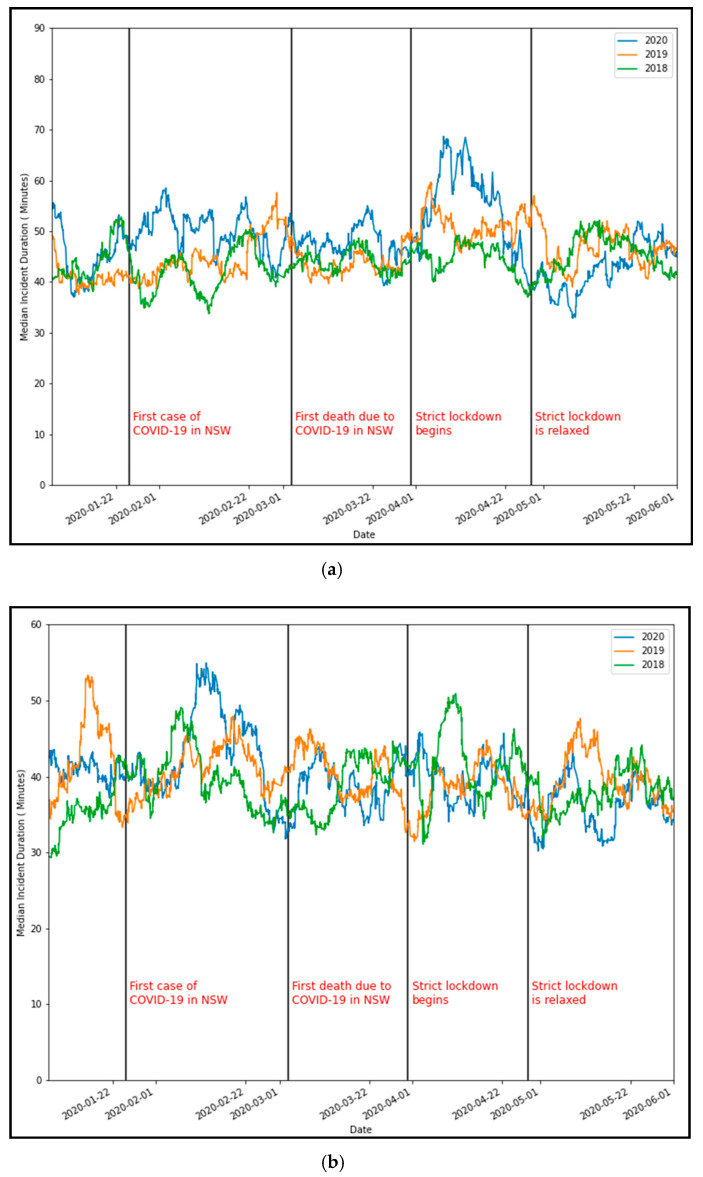
Comparison of seven-day rolling mean traffic incident duration (2020 vs. 2018 and 2019). (**a**) Crash duration and (**b**) breakdown duration.

Figure 6a,b showcase the seven-day rolling mean duration for crashes and breakdowns recorded in Sydney, along with key dates for COVID-related events in NSW. It is seen that the first lockdown induced a significant change in the mean incident duration for crashes compared to the same period in previous years when there was no lockdown. However, after the lockdown orders are loosened towards the end of the April, mean incident duration can be seen at its lowest point in three years. On the other hand, no discernible trend can be seen in the breakdown duration during the first lockdown.

**Figure 7:**
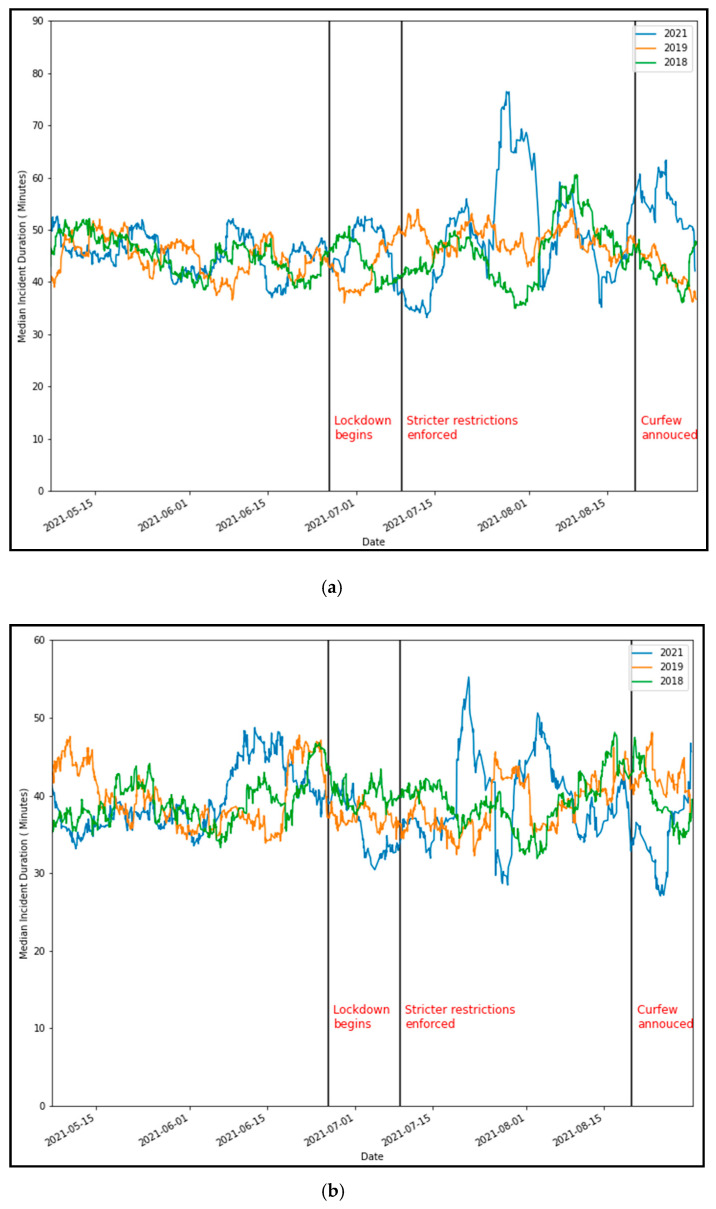
Comparison of seven-day rolling mean traffic incident duration (2021 vs 2018 and 2019). (**a**) Crash duration and (**b**) breakdown duration.

For the 2021 lockdown, we also see an increase in the duration of crashes for a certain period, most notably from the last week of July to the first week of August 2021 (Figure 7a). The trend for the breakdown duration is also mostly similar, apart from a decrease in the second half of August (Figure 7b).

As seen in Table 4, the mean crash duration increased by 16% during the first lockdown (*p*-value = 0.03). The breakdown duration decreased by 3%; however, it was statistically insignificant. The direction of the changes in duration was consistent in the second lockdown; however, they are statistically significant. An opportunity for analysis could be to explore further the impact of lockdown compliance with the changes in incident duration as Sydney saw increased anti-lockdown sentiment, with protests being prominent during the second lockdown [44,45].

Crashes involving diversions, emergency services and towing saw an increase in the mean duration from 2018/2019 to 2020. However, this was not reflected in the 2021 data, with only the major crashes seeing a significant increase. As these incident characteristics often reflect more severe incidents, this suggests that incidents became more severe during the first lockdown as individuals began to adjust their driving behaviour. Finally, none of the breakdown categories saw a significant change in duration during both of the lockdowns. Only the breakdowns involved in towing were reduced in duration during the second lockdown, with the *p*-value (0.08) close to being significant.

## 6. Conclusions

The impact of COVID-19 has been felt throughout the world. As a public crisis that has had devastating ramifications, there is significant evidence suggesting a shift in mobility patterns, and subsequently, a possible shift in driving behaviour and safety characteristics.

In the case of Sydney, there have been positive outcomes with a clear decrease in total crash and vehicle breakdown frequency during the lockdown periods. The decrease in crashes to the tune of 50–60% is consistent with most of the existing studies in Spain, Greece, Saudi Arabia and the USA [15,16,17,18,19]. However, characteristics that indicate more severe incidents (such as incidents labelled as major, fatal and/or involving towing) have increased proportionately, as the decreases have not been as significant as the overall incident rate. This observation is in line with studies done in Missouri and Connecticut in the USA.

With regard to crash duration, we observed a 16% increase during the first lockdown but no significant change during the subsequent lockdown. One can hypothesise that incident duration maybe shorter during the lockdowns as the attending groups no longer have to sit through peak hour traffic because of the overall reduction in congestion and traffic volumes. The alternative hypothesis is that because of the increased incident severity, it is challenging for the attending agencies to clear the incident quickly. The latter hypothesis was likely more pronounced during the first lockdown, while the second lockdown had a mix of both, thus leading to an insignificant change in overall duration.

There are various opportunities for further research. As COVID-19 begins to settle in, studying the effects of mobility changes and permanent shifts in travel patterns and incident characteristics, if any, would be appropriate. It would be interesting to analyse the spatial and temporal shifts in traffic incident hotspots and duration and their relationship with the socio-economic characteristics.

A key limitation within our study is the data source that was used. The data provided a wide range of information that is used for traveller information through NSW’s live traffic website. However, there is a lack of information regarding more specific incident characteristics such as the exact emergency services that attended, the speed at which the incidents took place, traffic flow conditions, etc. Furthermore, Sydney’s first lockdown was relatively short compared to other countries with multiple stages of mobility restrictions and health orders. Compiling a longer and more comprehensive dataset involving lockdowns with similar restrictions in different areas such as the wider Australia region would be advantageous, as the impact of different policies and various measures can be studied in relation to their impact on mobility and road traffic incidents. This is due to the evolving nature of mobility restrictions seen in Sydney, particularly with new information and restrictions on mobility often changing every week.

As such, COVID-19 reveals both challenges and opportunities for policy makers to design initiatives that aim to reduce future traffic demands, while also considering the impacts and ramifications on incident characteristics and ways to minimise this. We hope COVID-19 provides insight into what a future mobility network focused on sustainable and public transit solutions would be able to achieve for road safety.

## Figures and Tables

**Table 1:** Crash fatality in Sydney during the first lockdown compared to the corresponding period from previous years.

	Crash Fatality– First Lockdown Comparison		Crash Fatality– Second Lockdown Comparison	
	April 2020 (First Lockdown)	Average of 2018 and 2019	26 June 2021– 31 August 2021	Average of 2018 and 2019
Total Fatalities	7	10	8	17
Percentage of Fatalities Involving:				
Single Vehicle	0.57	0.50	0.63	0.59
Multiple Vehicles	0.43	0.50	0.38	0.41
Highways	0.71	0.20	0.25	0.21
Heavy Vehicles	0.43	0.25	0.25	0.15
Pedestrian	0.29	0.35	-	-

**Table 2:** Ratio of incident characteristics to the total incidents recorded for the month of April in 2020 (first lockdown), 2019 and 2018.

	2020		2019 and 2018			
	Total	Proportion	Average	Proportion	IRR	χ^2^ Statistic
**Total Incidents Recorded**	598		1198			
**Crashes**	273	0.4565	559	0.4664	0.98	0.19
**Breakdowns**	325	0.5435	639	0.5336	1.02	0.19
**Crashes involving:**						
Fatalities	7	0.0117	10	0.0084	1.40	0.60
Major incidents	18	0.0301	34	0.0284	1.06	0.05
Diversions	19	0.0318	23	0.0118	**1.69**	**3.85**
Heavy vehicles	59	0.0987	93	0.0776	1.27	2.91
Pedestrian	6	0.0100	16	0.0134	0.75	0.42
Motorcycle	22	0.0368	55	0.0459	0.80	0.95
Emergency services	242	0.4047	466	0.3887	1.04	0.51
Towing	53	0.0886	67	0.0555	**1.60**	**4.57**
Bicycles	10	0.0167	14	0.0112	1.48	1.16
**Breakdowns involving:**						
Major incidents	2	0.0033	9	0.0071	0.47	1.07
Diversions	2	0.0033	3	0.0025	1.34	0.13
Heavy vehicles	126	0.2107	238	0.1982	1.06	0.46
Motorcycle	3	0.0050	6	0.0050	1.00	0.00
Emergency services	99	0.1656	173	0.1441	1.15	1.75
Towing	52	0.0870	119	0.0994	0.88	0.84

Chi-square test statistic for 1 degree of freedom. χ^2^ > 3.841 for *p* < 0.05. The number in red (and bold) indicates that the IRR has increased during the lockdown period and it is statistically significant.

**Table 3:** Ratio of incident characteristics to the total incidents recorded for the period of 26 June to 31 August in 2021, 2019 and 2018.

	2021		2019 and 2018			
	**Total**	**Proportion**	**Average**	**Proportion**	**IRR**	**χ^2^ Statistic**
Total Incidents Recorded	1208		2854			
**Crashes**	519	0.4296	1461	0.5118	**0.84**	**26.94**
**Breakdowns**	689	0.5704	1393	0.4882	**1.17**	**26.94**
**Crashes involving:**						
Fatalities	8	0.0066	17	0.0060	1.11	0.07
Major incidents	15	0.0124	57	0.0198	0.63	2.99
Diversions	26	0.0215	57	0.0198	1.09	0.03
Heavy vehicles	83	0.0687	204	0.0715	0.96	0.12
Pedestrian	20	0.0166	48	0.0166	0.99	0.56
Motorcycle	42	0.0348	97	0.0340	1.02	0.02
Emergency services	439	0.3634	1183	0.4144	**0.88**	**10.75**
Towing	123	0.1018	162	0.0568	**1.79**	**33.46**
Bicycles	11	0.0091	10	0.0033	**2.74**	**7.70**
**Breakdowns involving:**						
Major incidents	0	0	11	0.0039	-	**4.67**
Diversions	1	0.0008	3	0.0011	0.79	0.05
Heavy vehicles	254	0.2103	534	0.1871	1.12	3.45
Motorcycle	5	0.0041	15	0.0053	0.79	0.25
Emergency services	189	0.1507	391	0.1370	1.10	3.12
Towing	148	0.1225	266	0.0932	**1.31**	**9.65**

Chi-square test statistic for 1 degree of freedom. χ^2^ > 3.841 for *p* < 0.05. The number in red (and bold) indicates that the IRR has increased during the lockdown period and it is statistically significant. The number in green (and bold) indicates that the IRR has decreased during the lockdown period and it is statistically significant.

**Table 4:** Comparison of mean incident durations for various incident types.

	Mean Incident Duration During the First Lockdown				Mean Incident Duration During the Second Lockdown			
	**2020**	**2019/2018**	**% Change**	***p*-Value**	**2021**	**2019/2018**	**% Change**	***p*-Value**
**Crashes**	54.69	47.01	**16%**	**0.03**	48.01	45.42	6%	0.32
**Breakdowns**	39.16	40.47	−3%	0.63	22.87	25.13	−9%	0.57
**Crashes involving:**								
Fatalities	-	-	-	-	-	-		-
Major incidents	170.50	123.84	38%	0.12	204.51	129.51	**58%**	**0.03**
Diversions	172.38	102.95	**67%**	**0.03**	107.82	137.63	−22%	0.33
Heavy vehicles	79.02	62.93	26%	0.19	71.86	63.8	13%	0.48
Pedestrian	44.11	63.85	−31%	0.5	76.4	63.23	21%	0.54
Motorcycle	73.00	45.31	61%	0.14	52.29	49.96	5%	0.93
Emergency services	57.83	49.88	**16%**	**0.04**	51.24	47.97	7%	0.32
Towing	88.66	60.14	**47%**	**0.02**	57.36	60.54	−5%	0.71
Bicycle	89.00	34.96	155%	0.09	28.99	34.09	−15%	0.86
**Breakdowns involving:**								
Major incidents	59.89	80.46	−26%	0.65	-	91.15		-
Diversions	34.44	94.71	−64%	0.35	85.57	46.96	82%	0.34
Heavy vehicles	63.56	61.8	3%	0.77	56.93	59.59	−4%	0.49
Motorcycle	14.11	13.24	7%	0.84	35.06	19.47	80%	0.31
Emergency services	36.06	41.91	−14%	0.63	39.87	42.54	−6%	0.57
Towing	41.89	33.58	25%	0.33	44.7	53.18	−16%	0.08

The number in red (and bold) indicates that the mean incident duration has increased during the lockdown period and it is statistically significant.

## Data Availability

The data presented in this study are available on request from the corresponding author.
